# A Ferroptosis-Related Gene Prognostic Index to Predict Temozolomide Sensitivity and Immune Checkpoint Inhibitor Response for Glioma

**DOI:** 10.3389/fcell.2021.812422

**Published:** 2022-01-31

**Authors:** Yonghua Cai, Xianqiu Liang, Zhengming Zhan, Yu Zeng, Jie Lin, Anqi Xu, Shuaishuai Xue, Wei Xu, Peng Chai, Yangqi Mao, Zibin Song, Lei Han, Jianqi Xiao, Ye Song, Xian Zhang

**Affiliations:** ^1^ Department of Neurosurgery, Nanfang Hospital, Southern Medical University, Guangzhou, China; ^2^ Department of Neurosurgery, The First Hospital of Qiqihar City, Qiqihar, China; ^3^ Department of Neurosurgery, Ganzhou People’s Hospital, Ganzhou, China

**Keywords:** ferroptosis, glioma, temozolomide, immune checkpoint inhibitor, tumor microenvironment, ferroptosis-based anticancer therapy, immunotherapy

## Abstract

**Background:** Gliomas are highly lethal brain tumors. Despite multimodality therapy with surgery, radiotherapy, chemotherapy, and immunotherapy, glioma prognosis remains poor. Ferroptosis is a crucial tumor suppressor mechanism that has been proven to be effective in anticancer therapy. However, the implications of ferroptosis on the clinical prognosis, chemotherapy, and immune checkpoint inhibitor (ICI) therapy for patients with glioma still need elucidation.

**Methods:** Consensus clustering revealed two distinct ferroptosis-related subtypes based on the Cancer Genome Atlas (TCGA) glioma dataset (*n* = 663). Subsequently, the ferroptosis-related gene prognostic index (FRGPI) was constructed by weighted gene co-expression network analysis (WGCNA) and “stepAIC” algorithms and validated with the Chinese Glioma Genome Atlas (CGGA) dataset (*n* = 404). Subsequently, the correlation among clinical, molecular, and immune features and FRGPI was analyzed. Next, the temozolomide sensitivity and ICI response for glioma were predicted using the “pRRophetic” and “TIDE” algorithms, respectively. Finally, candidate small molecular drugs were defined using the connectivity map database based on FRGPI.

**Results:** The FRGPI was established based on the *HMOX1*, *TFRC*, *JUN*, and *SOCS1* genes. The distribution of FRGPI varied significantly among the different ferroptosis-related subtypes. Patients with high FRGPI had a worse overall prognosis than patients with low FRGPI, consistent with the results in the CGGA dataset. The final results showed that high FRGPI was characterized by more aggressive phenotypes, high *PD-L1* expression, high tumor mutational burden score, and enhanced temozolomide sensitivity; low FRGPI was associated with less aggressive phenotypes, high microsatellite instability score, and stronger response to immune checkpoint blockade. In addition, the infiltration of memory resting CD4^+^ T cells, regulatory T cells, M1 macrophages, M2 macrophages, and neutrophils was positively correlated with FRGPI. In contrast, plasma B cells and naïve CD4^+^ T cells were negatively correlated. A total of 15 potential small molecule compounds (such as depactin, physostigmine, and phenacetin) were identified.

**Conclusion:** FRGPI is a promising gene panel for predicting the prognosis, immune characteristics, temozolomide sensitivity, and ICI response in patients with glioma.

## Introduction

Gliomas, including low grade (LGG, World Health Organization grades I–II) and high grade (HGG, World Health Organization grades III–IV), are the most common and lethal solid tumors of all brain cancers (Louis et al., 2016; Aldape et al., 2019). The annual incidence of gliomas is approximately six out of 100,000 individuals worldwide (Weller et al., 2021). Current glioma multimodal therapy includes surgery, radiotherapy, chemotherapy, and immunotherapy. Studies have identified IDH1 mutations, 1p19q chromosome deletions, and O-6-Methylguanine-DNA Methyltransferase (MGMT) epigenetic alterations as specific targets for enhancing treatment response, improving survival, and personalizing anticancer therapeutics (Nagashima et al., 2016). However, the overall prognosis remains poor, especially for glioblastomas, the most fatal of gliomas, which have 14.6 months of median survival (Tan et al., 2020). Additionally, there is 2- to 4-fold longer median survival in patients with IDH1 mutant glioma compared to those with IDH1 wild-type glioma (Yan et al., 2009; Nagashima et al., 2016).

Immune checkpoint inhibitors (ICI) have transformed the cancer treatment landscape, significantly improving survival (Brahmer et al., 2015; Larkin et al., 2015; Motzer et al., 2015; Bellmunt et al., 2017). Several studies have been conducted on immune-based therapy for gliomas, specifically for glioblastomas (Khasraw et al., 2020; Maire et al., 2020). Research has shown that standard therapies, including surgery, radiotherapy, and chemotherapy, may have immunosuppressive effects, further emphasizing opportunities to target the immune response for novel therapies (Lim et al., 2018). However, the initial results for trials of ICI, predominantly anti-*PD-1*/*PD-L1* and anti-*CTLA-4*, have been disappointing, and a phase III trial of nivolumab versus bevacizumab for recurrent glioblastoma did not demonstrate any meaningful benefits (Lim et al., 2018; Jackson et al., 2019; McGranahan et al., 2019; Reardon et al., 2020). Temozolomide, an imidazotetrazine prodrug alkylating agent, is an oral chemotherapeutic drug that crosses the blood–brain barrier and is widely used in patients with glioma (Friedman et al., 2000). Although temozolomide is traditionally considered to have a direct antitumor effect, many studies have confirmed its immunomodulatory properties (Karachi et al., 2018). However, approximately 55% of the patients with glioblastoma present with temozolomide resistance because of high MGMT expression (Hegi et al., 2005; Donson et al., 2007; van Nifterik et al., 2010). The evasion of apoptosis is, to a large extent, one of the main obstacles leading to poor therapeutic effects in malignant glioma. Thus, bypassing apoptosis resistance is a promising approach to overcome this problem (Gong et al., 2019; Tang et al., 2021).

Ferroptosis is a non-apoptotic cell death mechanism defined as iron-dependent regulated necrosis induced by membrane rupture mediated by excessive lipid peroxidation (Chen et al., 2021; Jiang et al., 2021). Ferroptosis has been proven to be a crucial tumor suppressor mechanism and may influence chemotherapy effects by triggering immune responses (Angeli et al., 2019). Many studies have shown that the expression of ferroptosis-related genes (including *GPX4, SLC7A11, and ACSL4*) is associated with sensitivity to temozolomide in glioma cells (Hu et al., 2020). In addition, T cells and interferon-gamma (IFN-γ) sensitize cancer cells to ferroptosis. Subsequently, the damage-associated molecular patterns (such as high mobility group box 1, *HMGB1*) released by cancer cells undergoing ferroptosis may induce cancer cell immunogenicity. This suggests that the role of ferroptosis in cancer therapy has synergistic potential with immunotherapy (Angeli et al., 2019; Liang et al., 2019; Llabani et al., 2019; Stockwell and Jiang, 2019; Wen et al., 2019; Tang et al., 2020). Ferroptosis-resistant cancer cells do not respond to *PD-L1* inhibitors, and the suppression of ferroptosis prevents gaining benefits from *PD-L1* inhibitors (Wang et al., 2019). Direct evidence of the link between ferroptosis and antitumor immunity was not evident until Wang et al. demonstrated that immunotherapy-activated CD8^+^ T cells suppressed tumor growth by sensitizing tumors to ferroptosis via IFN-γ (Wang et al., 2019; Tang et al., 2020). *In vitro* culture with low cystine and *in vivo* data showed that ferroptosis was involved in T-cell-mediated cancer immunity (Wang et al., 2019). Moreover, Liu et al. developed the ferroptosis potential index (FPI) to explore the functional roles of ferroptosis in various cancers, revealing that ferroptosis was associated with survival, the immune system, and chemotherapy resistance (Liu et al., 2020). Unfortunately, there is no ferroptosis-related potential prognostic marker that can simultaneously predict immune characteristics, temozolomide sensitivity, and the response of glioma to ICI.

In this study, we focused on the effects of ferroptosis on the immune characteristics of glioma and aimed to find markers that can accurately predict survival and ICI response of all patients with glioma. We screened hub ferroptosis-related genes (FRGs) by consistency analysis, WGCNA, and “stepAIC” algorithms to construct a ferroptosis-related gene prognostic index (FRGPI). We examined the prognostic predictive ability of FRGPI and characterized its molecular and immune profiles. We then predicted the sensitivity of patients with different FRGPI to temozolomide via the Genomics of Drug Sensitivity in Cancer (GDSC) database and the “pRRophetic” algorithms. Furthermore, we explored the relationship between the FRGPI and ICI responses through “TIDE” algorithms and verified it in an ICI therapy cohort.

## Materials and Methods

### Collection of Glioma Datasets, Ferroptosis-Related Genes (FRGs) and Immune-Related Gene (IRGs)

RNA sequencing (RNA-seq) and the corresponding complete clinical information for patients with glioma were retrieved from The Cancer Genome Atlas (TCGA) dataset (Version: 28.0, https://portal.gdc.cancer.gov/) and the Chinese Glioma Genome Atlas (CGGA) dataset (2021 Feb, http://www.cgga.org.cn/index.jsp) (Zhao et al., 2021). The main study was conducted using TCGA dataset (*n* = 663, including 248 LGG, 414 HGG, 1 unknown), and the CGGA dataset (*n* = 404, including 130 LGG and 274 HGG) was used for validation of the FRGPI for prognosis. The data of normal tissue samples were obtained from TCGA (*n* = 5), CGGA (*n* = 20), and GTEx (*n* = 2,642) (Version: 8.0, https://gtexportal.org/home/datasets). The gene-level transcription values were normalized to fragments per kilobase million (FPKM) and further transformed to log2 (FPKM+1) for downstream analysis. In addition, we used the “ComBat” function in the “sva” R package for the batcheffect within TCGA and GTEx datasets. Both somatic mutation data and copy number alteration data were obtained from TCGA database. The baseline clinical characteristics of glioma patients are shown in Supplementary Table 1. Twenty-four FRGs (Supplementary Table 2) were identified according to previously published literature (Liu et al., 2020). A total of 173 FRGs (Supplementary Table 3) were acquired from FerrDb (Version:1.0, http://www.zhounan.org/ferrdb/) (Zhou and Bao, 2020). A total of 1,793 IRGs (Supplementary Table 4) were obtained from ImmPort (https://www.immport.org/home) (Bhattacharya et al., 2018).

### Cell Culture

Human glioma cell lines U87-MG (HTB-14) and T98G (CRL-1690) were purchased from American Type Culture Collection (ATCC, Manassas, VA, United States). Human glioma cell lines were cultured in Dulbecco’s modified Eagle’s medium (DMEM; 06-1055-57-1A, Biological Industries) supplemented with 10% fetal bovine serum (FBS; 04-001-1A, Biological Industries) at 37°C with 5% CO_2_. The sources of all the cell lines were identified and verified by STR profiling. No *mycoplasma* contamination was detected in any of the cell lines by MycoSEQ™ *Mycoplasma* Real-Time PCR Detection Kit (ThermoFisher, 4460623).

### CCK-8 Assay

We used a CCK-8 kit (Beyotime, C0039) to measure the proliferation of U87-MG and T98G cells. First, we measured the IC_50_ of erastin, a ferroptosis activator. A total of 1 × 10^3^ cells (100 μl per well) were cultured in four replicate wells of a 96-well plate in a medium containing 10% FBS for 24 h. Then, erastin at different concentrations (0, 5, 10, 20, and 40 μmol/L) was added to a 96-well plate and divided into five groups (with an equal volume of DMSO added to the blank control group). Finally, the CCK-8 reagent (10 μl) was added to 90 μl DMEM to generate a working solution, of which 100 μl was added per well and incubated for 2 h. We performed this assay for 24 and 48 h after adding erastin. In U87-MG group, IC50 of erastin is 14.49 uM while 16.57 uM in T98 group. We then identified the influence of erastin on the proliferation ability of two glioma cell lines by adding different concentrations of temozolomide. All operations are roughly the same as the previous steps. The cells seeded in 96-well plates and were allowed to grow for 24 h. Then gradient concentrations of temozolomide and IC_50_ concentrations of erastin were added together in a 96-well plate. The drug concentrations of erastin (IC_50_ of U87 and T98G) at 24 h were too high, and this meant more DMSO had to be added, which had large impact on cell viability and proliferation both in the experimental and control groups and affected the accuracy of the experiment. Therefore, we chose 48 h as a reasonable drug treatment time. After 48 h, a 1:9 CCK reagent to DMEM was made and 100 µl of this solution was added to each well and incubated for 2 h.

### Immunofluorescence and Immunohistochemistry Staining

The subcellular distribution of proteins encoded by *GPX4*, *FSP1*, *GCH1*, and *DHODH* in human cell lines and their protein expression levels were investigated by confocal microscopy immunofluorescence (ICC-IF) and immunohistochemistry staining using the Human Protein Atlas database (Version: 21.0, https://www.proteinatlas.org/).

### Consensus Clustering for Patients With Glioma

The number of unsupervised classes in TCGA-Glioma dataset was estimated and validated based on the mRNA expression profiles of 24 FRGs (Supplementary Table 2) and 94 ferroptosis suppressors (Supplementary Table 5) using the consensus clustering method by the “ConsesusClusterPlus” R package.

### Somatic Mutation Landscape, stromalScore, immuneScore, and Immune Cell Infiltration in Distinct Ferroptosis-Related Subtypes

Mutation data were downloaded and visualized using the “maftools” R package to identify the somatic mutation landscape of patients with gliomas from TCGA database in distinct ferroptosis-related subtypes (Mayakonda et al., 2018). The immuneScore, stromalScore, and ESTIMATE score for each patient were calculated via the “estimate” R package (Yoshihara et al., 2013) and patients were divided into high and low immuneScore and stromalScore groups (based on the score above and below the median value, respectively). To make reliable immune infiltration estimations, we utilized the “immunedeconv” R package, which integrates six state-of-the-art algorithms, including TIMER, xCell, MCP-counter, CIBERSORT, EPIC, and quanTIseq (Sturm et al., 2019).

### Gene Set Enrichment Analysis and Gene Set Variation Analysis

Gene set enrichment analysis (GSEA) was conducted using the hallmark gene set “h.all.v7.2.symbols.gmt” between two ferroptosis-related subtypes using the “GSEA” R package. Gene set variation analysis (GSVA) was performed with the “GSVA” R package to compute a ssGSEA score of each functional pathway in each patient with glioma. The pathways with nominal *p*-value < 0.05, and false discovery rate (FDR) < 0.05, were considered significantly enriched.

### The Construction of FRGPI and Nomogram Model

Differentially expressed genes (DEGs) with FDR < 0.05 and |Fold Change (FC)| ≥ 1 were identified among different clusters and groups using the “limma” R package and visualized using volcano plots and heatmaps. Weighted gene co-expression network analysis (WGCNA) (Langfelder and Horvath, 2008) was performed using the “WGCNA” R package (networkType = “unsigned”, minModuleSize = 30) to identify modules associated with ferroptosis (correlation coefficient > 0.50 and *p*-value < 0.05 were set as the inclusion criterion). Protein–protein interaction (PPI) analysis was performed using STRING database (https://string-db.org/). The FRGPI was established using the “stepAIC” algorithm in the “MASS” R package, and the model with the lowest Akaike Information Criterion (AIC) value was then chosen as the best model to yield the FRGPI equation with the coefficient multiplied by mRNA expression. According to this equation, the FRGPI of each patient was calculated separately for the training and validation cohorts. Subsequently, the patients were divided into high and low FRGPI groups, and the median value of the FRGPI score was set as the cut-off point. Principal component analysis (PCA) was performed using these signature genes. Univariate and multivariate Cox regression analyses were performed to identify the appropriate terms to build the nomogram. A forest plot was used to show the *p*-value, hazard ratio (HR), and 95% confidence interval (CI) of each variable through the “forestplot” R package. The nomogram was built based on the multivariate cox proportional hazards analysis (“*p*-value < 0.05” was set as the inclusion criterion) through the “rms” R package to predict the 1-, 3-, and 5-year overall survival (OS).

### Estimation of Ferroptosis Potential Index, Tumor Mutational Burden, Microsatellite Instability, and Stemness Index

The FPI, which can be used to represent the potential level of ferroptosis based on transcriptome data, was computed for each sample according to previously published literature (Liu et al., 2020). Tumor mutational burden (TMB) score, microsatellite instability (MSI) score, and two independent stemness indices (mRNA expression-based stemness index, mRNAsi, and DNA methylation-based stemness index, mDNAsi) were calculated according to previously defined methods (Bonneville et al., 2017; Chalmers et al., 2017; Malta et al., 2018).

### Correlation of FRGPI With Temozolomide Sensitivity and ICI Response

The sensitivity of each patient to temozolomide was estimated using the GDSC database (https://www.cancerrxgene.org/). The estimated half-maximal inhibitory concentration (IC_50_) was quantified via the “pRRophetic” R package. The potential ICI response was predicted using the TIDE algorithm (Jiang et al., 2018). Furthermore, one immunotherapy cohort (metastatic urothelial cancer treated with atezolizumab, IMvigor210 cohort) (Mariathasan et al., 2018) was included to validate the tumor response to *PD-L1* blockade.

### Predicting Candidate Small Molecules Based on FRGPI

The DEGs (Supplementary Table 9) with FDR < 0.05 and |FC| ≥ 1 were identified as high- and low-FRGPI groups by the “limma” R package, and the function enrichment terms of DEGs were acquired from the STRING database (https://string-db.org/). Based on these DEGs, candidate small molecular drugs and mechanisms of action were defined using the connectivity map database (CMap, http://portals.broadinstitute.org/cmap/) (Lamb et al., 2006) and CMap mode-of-action analysis.

### Statistical Analysis

All statistical analyses were conducted using R software (version 4.0.2). The normality of the variables was tested using the Shapiro-Wilk normality test. For comparisons of two normally distributed groups, statistical analysis was performed using unpaired *t*-tests, and for non-normally distributed variables, statistical analysis was performed using the Wilcoxon rank-sum test. For comparisons of three or more groups, Kruskal-Wallis tests or one-way ANOVA were used as nonparametric or parametric methods, respectively. Correlations between normally distributed variables were assessed using Pearson’s correlation test, while correlations between non-normally distributed variables were assessed using Spearman’s correlation test. A *p*-value < 0.05, and |correlation coefficient |(R)| > 0.30 were considered as statistically significant. Kaplan–Meier curves for OS were presented between different subgroups, and OS comparisons were performed using the log-rank test. The receiver operating characteristic (ROC) curve was used to assess the prognosis classification performance of the FRGPI model, and the area under the curve (AUC) was calculated using the “timeROC” R package. All statistical *p* values were two-sided, with *p* < 0.05 considered statistically significant.

## Results

### Ferroptosis Plays a Potential Functional Role in the Progression and Treatment of Glioma

**FIGURE 1:**
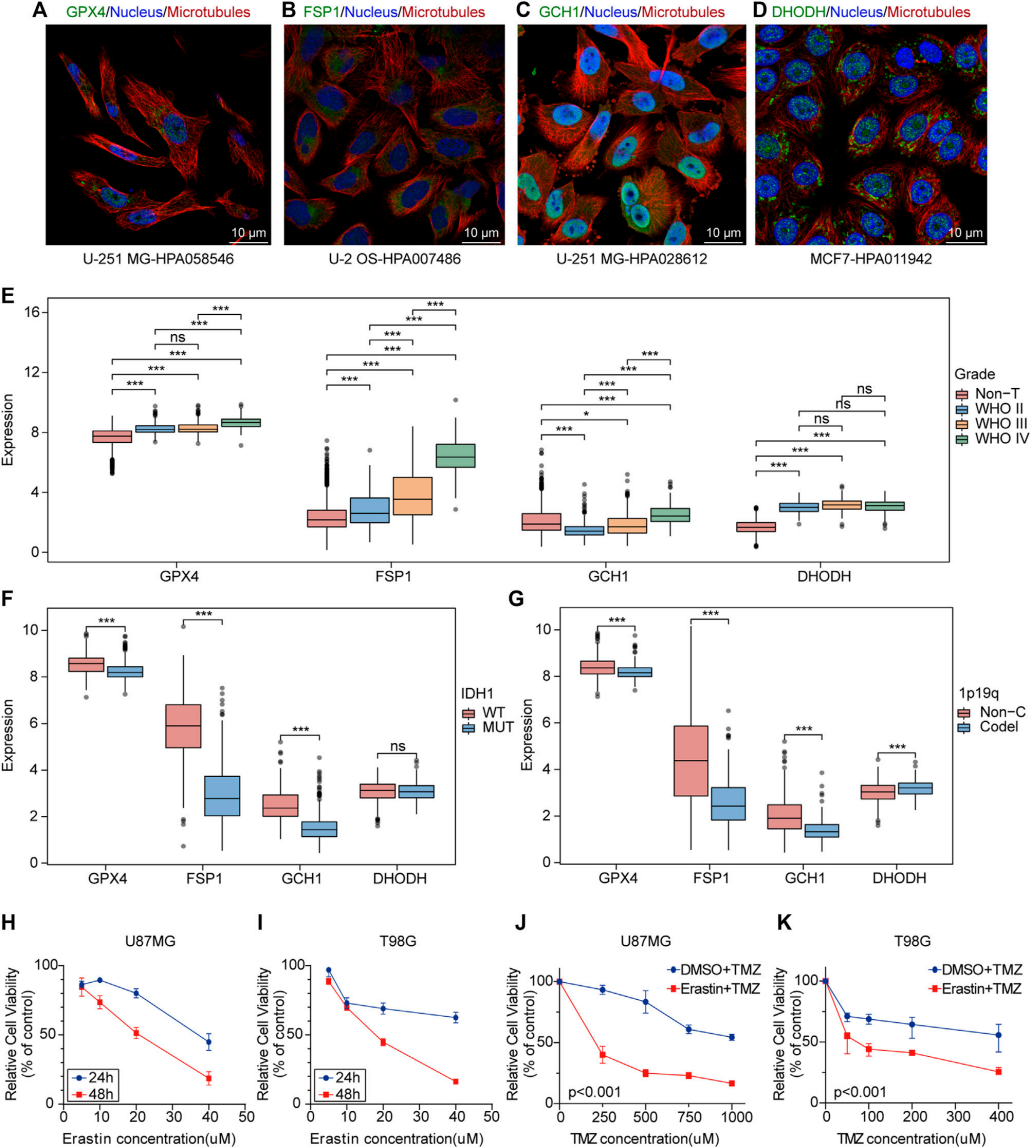
Ferroptosis plays an potential biological role in the progression and treatment of glioma. **(A–D)** Confocal immunofluorescence staining for *GPX4*, *DHODH*, *GCH1*, *FSP1* in cell lines from HPA database. Green, antibody; blue, nucleus; red, microtubules. **(E–G**) Boxplots of *GPX4*, *GCH1*, *FSP*1 and *DHODH* expression in different grade, IDH1-status and 1p19q-codeletion-status glioma samples from TCGA database. ns, no significance; *p < 0.05; **p < 0.01; ***p < 0.001. **(H,I)** The cell viability of U87MG and T98G cells transfected with erastin was evaluated by the CCK-8 assays in 24 and 48 h. Five technical repeats were performed for two biological repeats. **(G,K)** The cell viability of U87MG and T98G cells transfected with erastin and temozolomide was evaluated by the CCK8 assays in 48 h. Five technical repeats were performed for two biological repeats.

*GPX4*, *FSP1*, *GCH1*, and *DHODH* are key genes involved in cell ferroptosis defense (Zheng and Conrad, 2020; Mao et al., 2021). Immunofluorescence and confocal microscopy showed that *GPX4* was mainly located in nucleoplasm and mitochondria, *DHODH* was located in the mitochondria, *GCH1* was located in nucleoplasm and cytosol, and *FSP1* was located in cytosol (Figures 1A–D). The analysis of mRNA expression profiles of glioma samples and non-tumor brain tissues provided evidence that *GPX4*, *FSP1*, and *DHODH* were highly expressed in tumoral tissue compared to that in non-tumor brain tissues (Figure 1E and Supplementary Figures 1A,B). Additionally, the expression of *GCH1* and *FSP1* increased with increasing malignancy of the tumor (Figure 1E); *GPX4*, *FSP1,* and *GCH1* were highly expressed in patients with glioma and wild-type *IDH1* compared to mutant *IDH1* (Figure 1F). *GPX4*, *FSP1*, and *GCH1* were highly expressed, while *DHODH* expression was low in patients with gliomas with a 1p19q non-codeletion compared to those with a 1p19q codeletion (Figure 1G). Immunohistochemistry (IHC) demonstrated that the four genes were highly expressed at the protein level in gliomas compared to non-tumor brain tissues (Supplementary Figures 1C,D). The CCK-8 assay results showed that ferroptosis induction by erastin inhibited proliferation and enhanced the sensitivity of U87MG and T98G cells to temozolomide (Figures 1H–K). These data indicate that ferroptosis played an important role in the maintenance, progression, and treatment of gliomas.

### Clinical Features, Somatic Landscapes, and Immune Infiltration of Different Ferroptosis-Related Subtypes in Glioma

**FIGURE 2:**
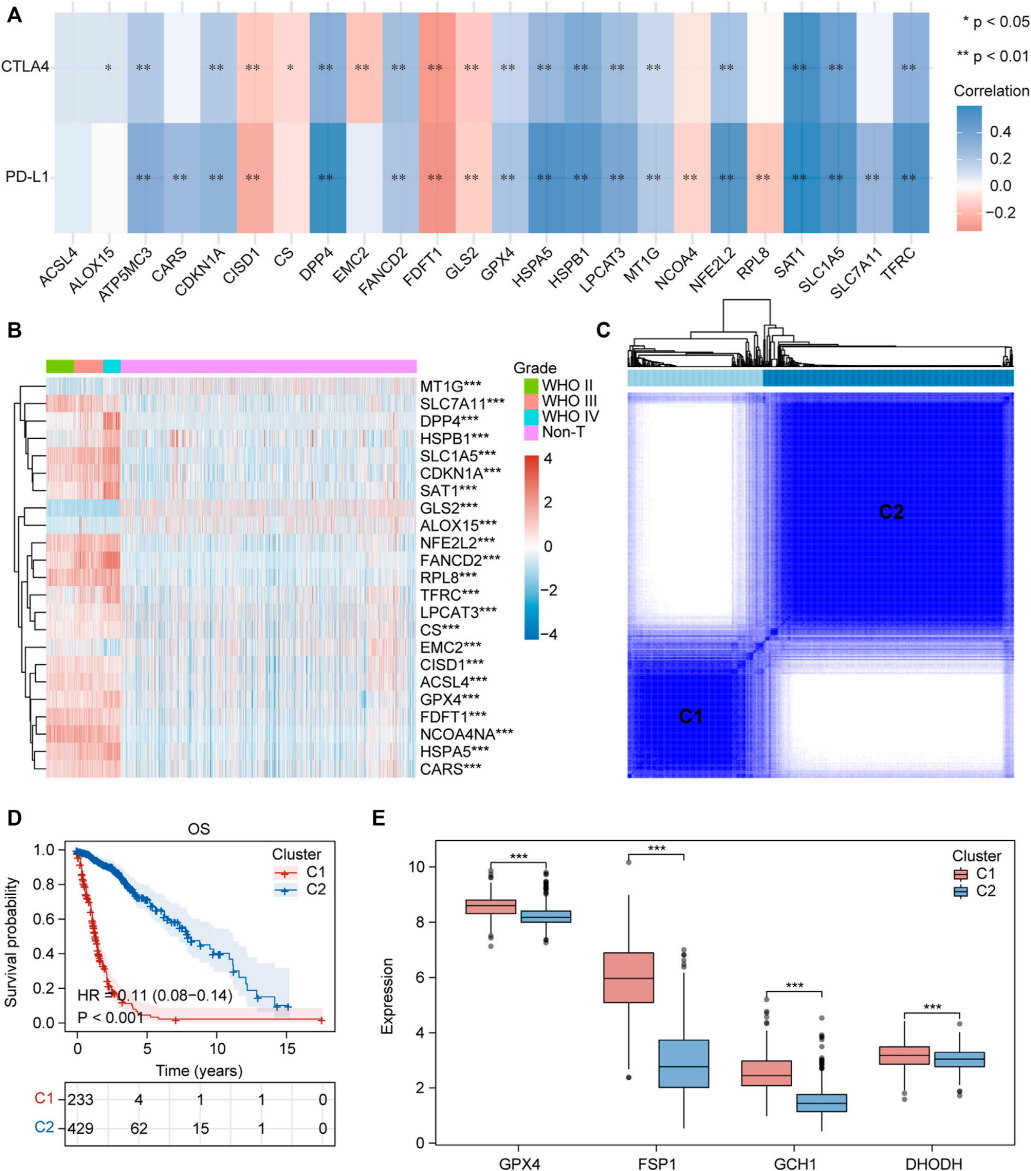
Two ferroptosis-related subtypes were identified based on the expression profile of FRGs. **(A)** Correlation heatmap between 24 FRGs expression and *PDL-L1*/*CTAL4*. Blue, positive correlation; red, negative correlation. *p < 0.05, **p < 0.01. **(B)** Heatmap depicted the FRGs expression profile landscape in gliomas of TCGA database. Red, high expression, blue, low expression. **(C)** Consensus clustering matrix for k = 2. **(D)** Kaplan–Meier analysis of patients in the two different ferroptosis-related subtypes. **(E)** Boxplots of *GPX4*, *GCH1*, *FSP1* and *DHODH* expression level in the two different ferroptosis-related subtypes. ns, no significance; *p < 0.05; **p < 0.01; ***p < 0.001.

**FIGURE 3:**
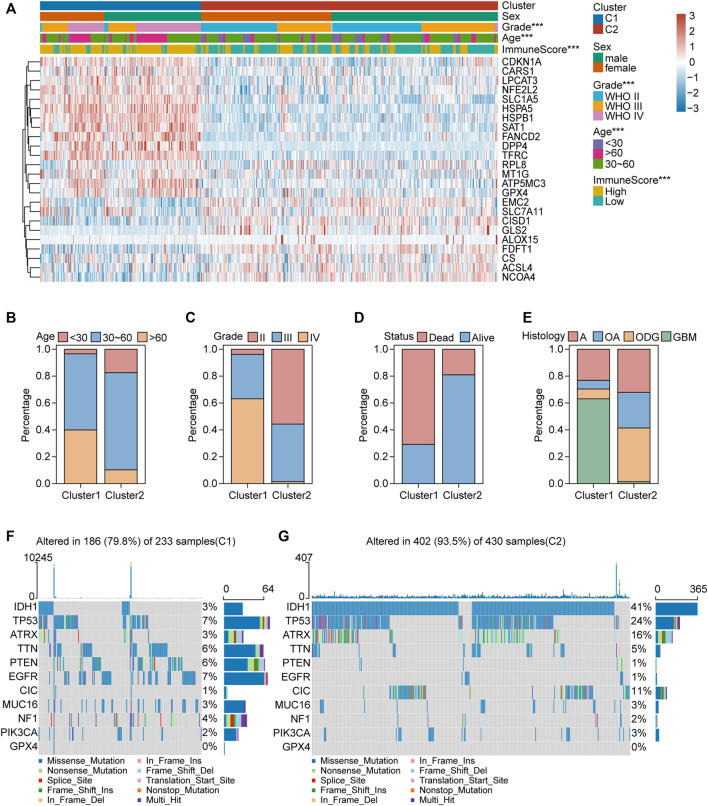
Clinical features and somatic landscapes of two ferroptosis-related subtypes. **(A)** Heatmap visualized the distribution of sex, age, grade, immuneScore between the two ferroptosis-related subtypes. *p < 0.05; **p < 0.01; ***p < 0.001. **(B–E)** Histograms depicted the differences of age, grade, status and histology between two ferroptosis-related subtypes. **(F,G)** Oncoplot displaying the somatic landscape of glioma cohort of two ferroptosis-related subtypes. Genes are ordered by their mutation frequency, and samples are ordered according to disease histology as indicated by the annotation bar (bottom). Side bar plot shows log10 transformed Q-values estimated by MutSigCV. Landscape of mutation profiles in OC samples. Mutation information of each gene in each sample was shown in the waterfall plot, where different colors with specific annotations at the bottom meant the various mutation types. The bar plot above the legend exhibited the number of mutation burden.

**FIGURE 4:**
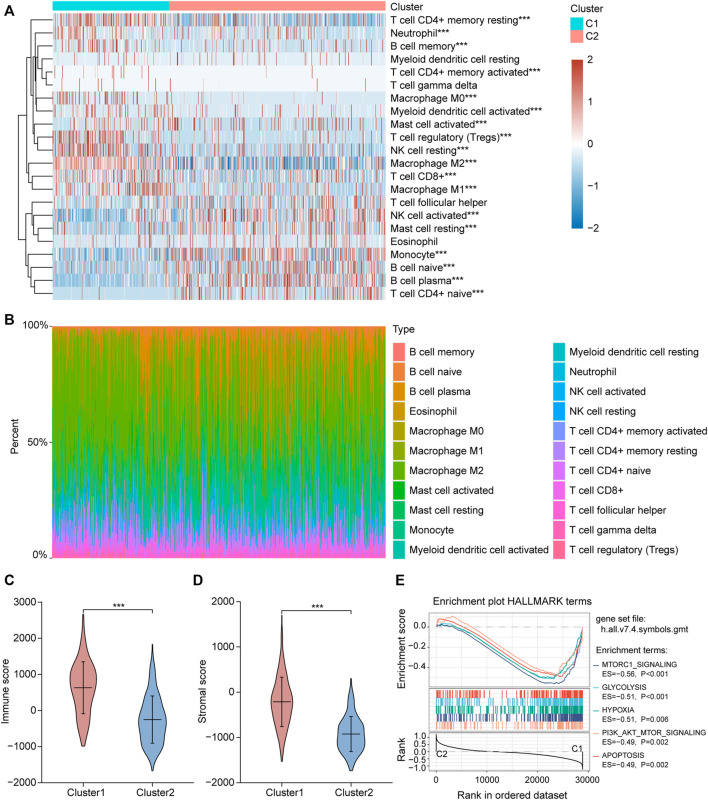
Different immune cell infiltration between two ferroptosis-related subtypes. **(A)** Immune cell score heat map, where different colors represent the expression trend in each sample between two ferroptosis-related subtypes. *p < 0.05, **p < 0.01, ***p < 0.001. **(B)** The percentage abundance of tumor infiltrating immune cells in each sample, with different colors and different types of immune cells. The abscissa represents the sample, and the ordinate represents the percentage of immune cell content in a single sample. **(C,D)** Violin plots of the immuneScore and stromalScore between the two ferroptosis-related subtypes. *p < 0.05, **p < 0.01, ***p < 0.001. **(E)** The enrichment plots of representative Gene Set Enrichment Analysis (GSEA) results. ES, enrichment score.

We collected 24 FRGs according to Liu et al. (2020). Spearman correlation analysis indicated a significant correlation between the mRNA expression of FRGs and *PDL-L1/CTAL4* (Figure 2A). Most FRGs were highly expressed in glioma tissues compared to non-tumor brain tissues, as shown in Figure 2B. The patients with glioma were divided into two ferroptosis-related subtypes (C1 and C2) by performing consistency analysis based on FRGs mRNA expression profiles (Figure 2C). The OS for patients in C1 was shorter than that for patients in C2 (Figure 2D). *GPX4*, *GCH1*, *FSP1*, and *DHODH* were expressed at higher levels in patients in C1 than in C2 (Figure 2E). The expression of FRGs and clinical features of the two subtypes were then compared (Figure 3A). The C1 group mainly contained patients with glioma over 60 years of age, preferentially associated with a high WHO grade and glioblastoma (Figures 3B–E). The oncoplot displaying the somatic landscape showed that *IDH1*, *TP53*, *ATRX*, and *CIC* mutation frequencies in C2 were higher than in C1, but *PTEN* and *EGFR* in C1 were higher than in C2 (Figures 3F,G). A total of 22 immune cell infiltration scores of each sample were estimated using the CIBERSORT algorithm. The scores of resting and activated memory CD4^+^ T cells, CD8^+^ T cells, regulatory T cells (Tregs), neutrophils, memory B cells, M0 macrophages, M1 macrophages, M2 macrophages, activated myeloid dendritic cells, and activated mast cells in the samples of C1 were higher than those of C2. However, the activated NK cell, resting mast cell, monocyte, naïve B cell, plasma B cell, and naïve CD4^+^ T cell scores in the samples of C1 were lower than in those in C2 (Figure 4A). The percentage of tumor-infiltrating immune cells in each sample is shown in Figure 4B. The ESTIMATE algorithm showed that the immuneScore and stromalScore in the samples of C1 were higher than those of C2 (Figures 4C,D). To elucidate the underlying regulatory mechanisms leading to the difference between the two ferroptosis subtypes in glioma, GSEA was performed. Our data indicated that PI3K_AKT_MTOR_SIGNALING, MTORC1_SIGNALING, GLYCOLYSIS, HYPOXIA, and APOPTOSIS were mainly enriched in the C1 group but were not enriched in the C2 group (Figure 4E). These results suggest that distinct differences in the clinical, genomic, and immune infiltration characteristics of ferroptosis-related subtypes were divided by the expression of 24 FRGs.

To verify this hypothesis, consistency analysis was performed based on 94 ferroptosis suppressors that have been verified in humans and dividing the sample into ferroptosis subgroups (including G1 and G2; Supplementary Figure 2A). Survival analysis revealed that patients in G2 had longer OS than those in G1 (Supplementary Figure 2B). The differences in immuneScore, stromalScore, and tumor-infiltrating immune cell scores between G1 and G2 were similar to the differences between C1 and C2 (Supplementary Figures 2C–E).

### FRGPI Was Developed to Reveal the Functional Roles of Ferroptosis in Glioma Based on FRGs

**FIGURE 5:**
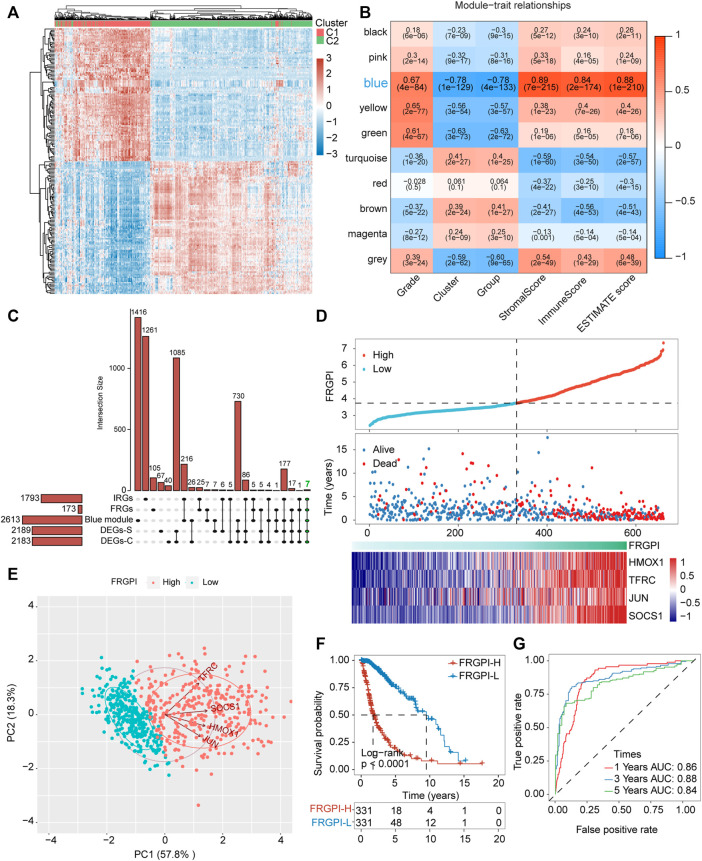
FRGPI was developed based on FRGs for glioma. **(A)** Differential gene expression heat map, where different colors represent expression trends in different tissues. Due to the large number of differential genes, the 50 up-regulated genes and 50 down-regulated genes with the largest differential changes are shown here. **(B)** Correlation of weighted gene correlation network analysis (WGCNA) modules with ferroptosis-related subtypes and immuneScore. **(C)** Upset plot for DEGs between the two ferroptosis-related subtypes, DEGs between the two ferroptosis-related subgroups, FRGs, IRGs and genes in blue module. IRGs, immune-related genes. **(D)** FRGPI, survival outcome and *HMOX1*, *TFRC*, *JUN* and *SOCS1* expression profiles of each sample are shown. **(E)** Principal component analysis (PCA) plot of glioma samples based on *HMOX1*, *TFRC*, *JUN* and *SOCS1* expression profiles. **(F)** Kaplan–Meier analysis of glioma patients with low or high FRGPI. **(G)** ROC curves predicted prognostic value of FRGPI in glioma patients.

Differential expression gene analysis was performed between C1 and C2 or G1 and G2, and the DEGs (Supplementary Tables S6, S7) expression heatmap is shown in Figure 5A and Supplementary Figure 3A. WGCNA was performed to divide all protein-coding genes into 10 modules (Supplementary Figure 3B), of which the one most related to ferroptosis-related subtypes and tumor immunity was the blue module (Figure 5B). Then, 173 FRGs were acquired from the FerrDb database, and 1,793 IRGs were obtained from the ImmPort database. Furthermore, FRGs, IRGs, DEGs, and genes in the blue module (Supplementary Table 8) were intersected, and seven hub genes (*JUN, TNFAIP3, NOX4, HMOX1, SOCS1, CYBB,* and *TFRC*), related to both ferroptosis and tumor immunity, were obtained (Figure 5C). The protein-protein interaction network of the seven hub genes is shown in Supplementary Figure 3C. The seven hub genes were used to develop the FRGPI model in TCGA training set using the stepAIC algorithm. The optimal model equation was yielded using four factors (*HMOX1, TFRC, JUN,* and *SOCS1*) with the lowest AIC (Figure 5D):

FRGPI=(0.279×HMOX1 expression level)+(0.558×TFRC expression level)+ (0.107×JUN expression level)+ (0.469 × SOCS1 expression level)

**FIGURE 6:**
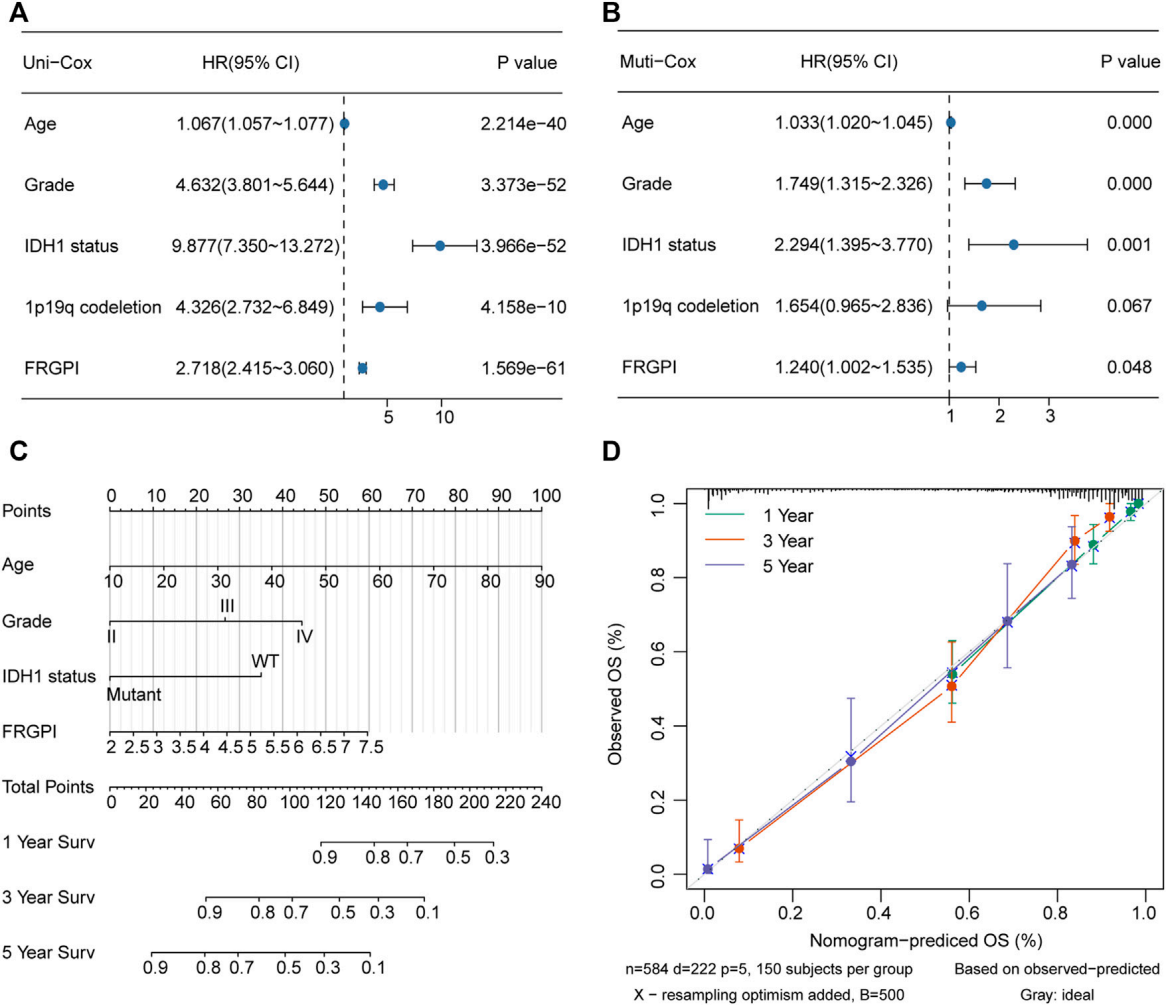
Nomogram was constructed to calculated prognostic risk score for individual. **(A,B)** Hazard ratio and *p*-value of constituents involved in univariate and multivariate Cox regression for FRGPI, age, grade, IDH1 status and 1p19q codeletion in TCGA glioma samples. **(C)** Nomogram to predict the 1-, 3- and 5-year OS of glioma patients. **(D)** Calibration curve indicated that predicted 1-, 3- and 5-year survival rates were close to the actual survival rates. The gray dashed diagonal line represents the ideal nomogram, and the green line, red line and blue line represent the 1-, 3- and 5-y observed nomograms.

The model was verified in the CGGA validation set (Supplementary Figure 4A). PCA verified that patients with glioma in TCGA training set or the CGGA validation set could be divided into high and low FRGPI groups, respectively (Figure 5E; Supplementary Figure 3D). Compared with low FRGPI, patients with glioma and a high FRGPI had a worse OS (Figure 5F; Supplementary Figure 4B), with a high predictive accuracy of FRGPI for OS (TCGA training set: 1 year AUC: 0.86, 3 years AUC: 0.88, 5 years AUC: 0.84; CGGA validation set: 1 year AUC: 0.60, 3 years AUC: 0.70, 5 years AUC: 0.70) (Figure 5G and Supplementary Figure 4C). Univariate and multivariate Cox regression analyses indicated that FRGPI was a variable independent of other clinical factors, including age, grade, *IDH1* status, and 1p19q codeletion status (Figures 6A,B). Additionally, the prediction accuracy of FRGPI was highly stable in different clinical character groups, including <45 and >45-year age group, LGG (grade I–II gliomas are classified as LGG in the TCGA database) and GBM (grade IV gliomas) groups, IDH1-WT and IDH1-Mut groups, 1p19q non-codel group, except in 1p19q codel group, in TCGA training set (Supplementary Figures 5A–H), and these results were verified again in the CGGA validation set (Supplementary Figures 5I–P). A nomogram was developed based on the multivariate Cox proportional hazards analysis results to precisely calculate the prognostic total risk points for an individual (Figure 6C). The calibration curve showed that the predicted 1-, 3-, and 5-year survival probabilities by the nomogram were close to the actual survival probability (Figure 6D).

### FRGPI Is Associated With Clinicopathologic Features, Immunity, and Intrinsic Immune Escape in Glioma

**FIGURE 7:**
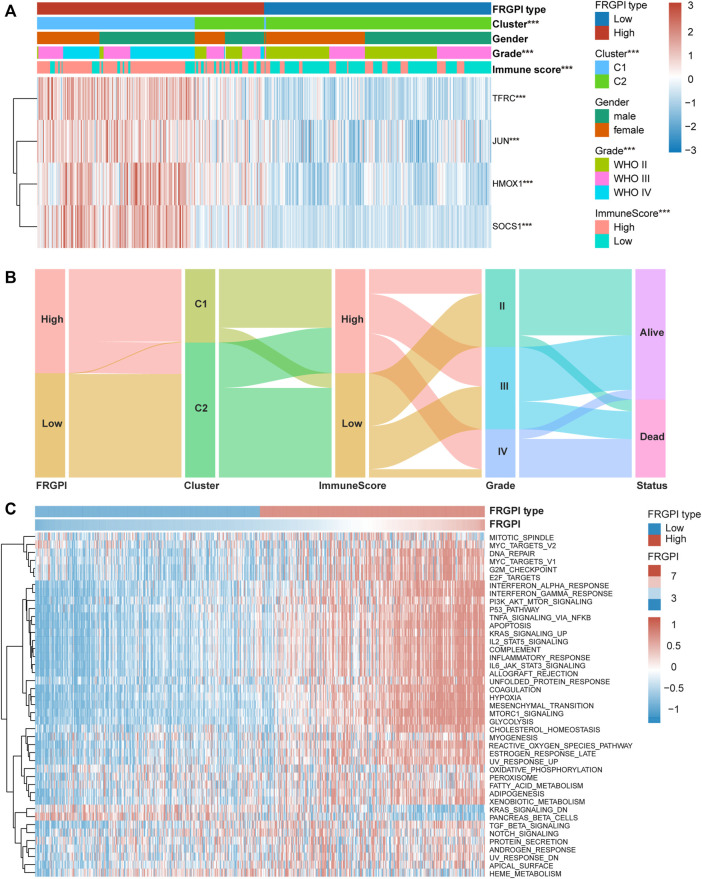
High FRGPI is associated with glioma progression. **(A)** Heatmap showed that ferroptosis-related subtypes, grade and immuneScore were significantly associated with FRGPI. *p < 0.05; **p < 0.01; ***p < 0.001. **(B)** The relationship between FRGPI, ferroptosis-related subtypes, immuneScore, grade and survival status in glioma patients was illustrated by the Sankey diagram. **(C)** Heatmap of Gene set variation analysis (GSVA) results between High and Low FRGPI groups.

**FIGURE 8:**
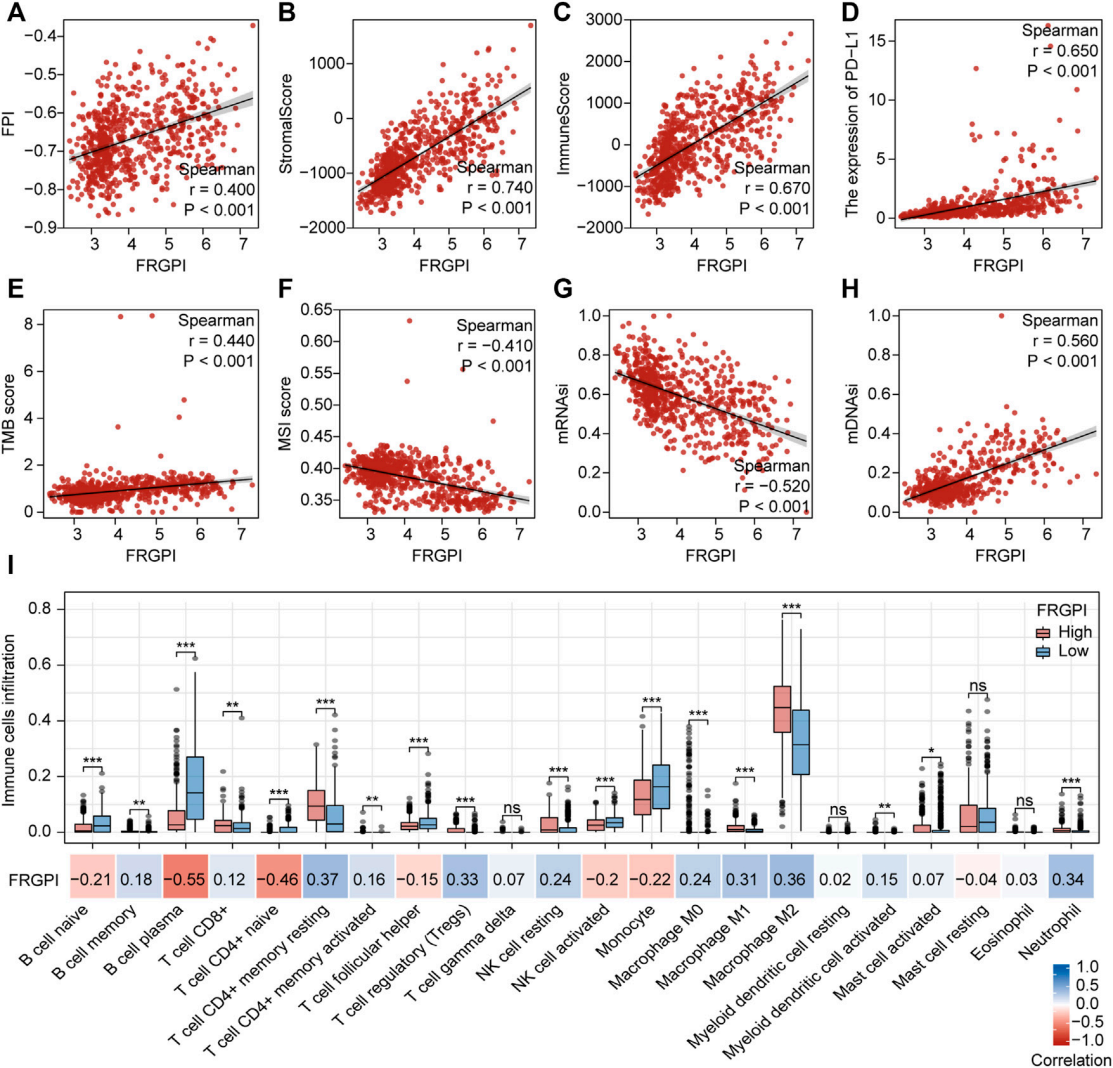
Anti-tumor immunity and intrinsic immune escape associated with FRGPI. Spearman correlation coefficients and associated *p*-value of FRGPI with **(A)** FPI, **(B)** stromalScore, **(C)** immuneScore, **(D)** the expression of *PD-L1*, **(E)** TMB score, **(F)** MSI score, **(G)** mRNAsi, **(H)** mDNAsi are shown. r, Spearman coefficient; ns, no significance; *p < 0.05; **p < 0.01; ***p < 0.001. **(I)** Different immune cell infiltration between high and low FRGPI group, and the correlation between FRGPI and immune cells infiltration. ns, no significance; *p < 0.05; **p < 0.01; ***p < 0.001; blue, positive correlation; red, negative correlation.

The expression of *TFRC*, *JUN*, *HMOX1*, and *SOCS1* and the clinicopathologic features between the two FRGPI groups were compared. *TFRC*, *JUN*, *HMOX1*, and *SOCS1* were highly expressed in the high FRGPI group. Most of the patients with glioma in C1, with high WHO grade or high immuneScore, were distributed in the high FRGPI group (Figure 7A). The Sankey diagram fully summarized the association between FRGPI, ferroptosis-related subtypes, clinical characteristics, and prognostic signature (Figure 7B). In addition, GSVA was conducted to assess potential changes in pathway activity, and the results showed that most of the pathways associated with cancer progression were activated in patients with glioma with high FRGPI (Figure 7C). The FPI was established to model ferroptosis levels by Liu et al. We found that FRGPI was positively correlated with FPI (Figure 8A), which implied that FRGPI could also be associated with antitumor immunity and intrinsic immune escape in glioma. Spearman correlation test was conducted between FRGPI and stromalScore, immuneScore, the expression of PD-L1, TMB score, and MSI score (Figures 8B–F). FRGPI positively correlated with the stromalScore (Spearman: r = 0.670, *p* < 0.001), immuneScore (Spearman: r = 0.670, *p* < 0.001), PD-L1 expression (Spearman: r = 0.650, *p* < 0.001), and TMB score (Spearman: r = 0.440, *p* < 0.001), but negatively correlated with MSI score (Spearman: r = –0.410, *p* < 0.001). Previous research has indicated potential associations between the tumor immune microenvironment and cancer cell stemness (Saygin et al., 2019). We calculated stemness indices (mRNAsi and mDNAsi) for each glioma sample according to Malta et al. (2018), and found that FRGPI was negatively correlated with the mRNAsi score (Spearman: r = –0.520, *p* < 0.001, Figure 8G), but positively correlated with the mDNAsi score (Spearman: r = 0.560, *p* < 0.001, Figure 8H). Twenty-two immune cell infiltration scores of the different FRGPI groups were compared, and the correlation between FRGPI and each immune infiltrating cell score was calculated using the Spearman correlation test (Figure 8I). The scores of resting memory T cells CD4^+^ (Spearman: r = 0.37, *p* < 0.001), regulatory T cells (Spearman: r = 0.33, *p* < 0.001), M1 macrophages (Spearman: r = 0.31, *p* < 0.001), M2 macrophages (Spearman: r = 0.36, *p* < 0.001), and neutrophils (Spearman: r = 0.34, *p* < 0.001) were positively correlated with FRGPI, while the scores of plasma B cell (Spearman: r = –0.55, *p* < 0.001), and naïve T cell CD4^+^ (Spearman: r = –0.46, *p* < 0.001) were negatively correlated with FRGPI. These results indicate that FRGPI was associated with antitumor immunity and intrinsic immune escape in glioma. The GSVA results (Supplementary Figures 3E,F) showed FRGPI was highly correlated with PI3K_AKT_MTOR_SIGNALING (Spearman: r = 0.660, *p* < 0.001) and MTORC1_SIGNALING (Spearman: r = 0.810, *p* < 0.001).

### A High FRGPI Is More Sensitive to Temozolomide, but a Low FRGPI Is More Sensitive to ICI Therapy

**FIGURE 9:**
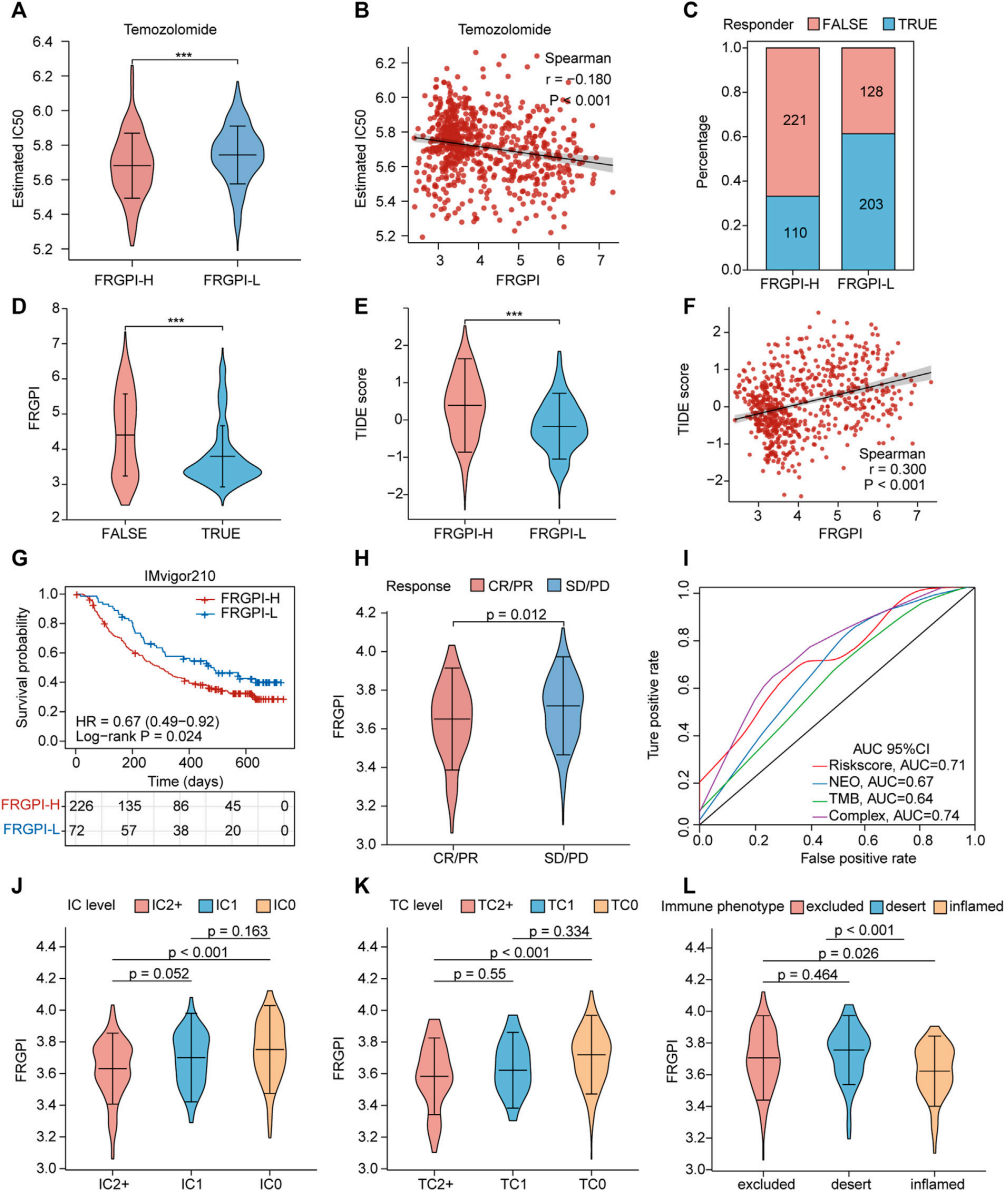
FRGPI is predictive of temozolomide sensitivity and ICI response in glioma. **(A)** Different estimated temozolomide IC_50_ between high and low FRGPI group. **(B)** The spearman correlation between FRGPI and estimated temozolomide IC_50_. The ICI response of each patient in high and low FRGPI group was predicted by TIDE algorithm, **(C)** stacked histogram showed the distribution of “TURE” or “FALSE” responder in high and low FRGPI group; **(D,E)** Violin plots showed the FRGPI level of “TURE”- and “FALSE”-responder group, and the different TIDE score between high- and low-FRGPI group. **(F)** The spearman correlation between FRGPI and TIDE score. **(G)** Kaplan–Meier curves showing overall survival in patients with low or high FRGPI in the anti-*PD-L1* cohort. **(H)** The distribution of FRGPI in distinct anti-*PD-L1* clinical response groups. **(I)** Roc curves showing predicting value of FRGPI, neoantigen (NEO), TMB and complex (FRGPI combined with NEO and TMB) group for anti-*PD-L1* therapy response. CR, complete response; PD, disease progression, PR, partial response; SD, stable disease. **(J–L)** Distribution of the FRGPI in distinct IC-level, TC-level and immune-phenotype group respectively in the anti-*PD-L1* cohort. IC-level, *PD-L1* expression level on immune cells; TC-level, *PD-L1* expression level on tumor cells.

Next, we explored the role of FRGPI in the treatment of glioma. We investigated if FRGPI predicts the sensitivity of gliomas to temozolomide chemotherapy. Our data suggested that the estimated IC_50_ of temozolomide in the high FRGPI group was significantly lower than that in the low FRGPI group (Figure 9A), and FRGPI was negatively correlated with the IC_50_ of temozolomide (Spearman: r = –0.180, *p* < 0.001; Figure 9B), suggesting that patients with glioma and high FRGPI were more sensitive to temozolomide therapy. Then, the correlation between FRGPI and the sensitivity of immunotherapy for glioma was explored. The potential ICI response was predicted using the TIDE algorithm. Our data suggested that the proportion of people responding to ICI therapy in the low FRGPI group was greater than that of the high FRGPI group (Figure 9C), and the “TURE-responder” group had significantly lower FRGPI than the “FALSE-responder” group (Figure 9D). The low FRGPI group had significantly lower TIDE scores than the high FRGPI group (Figure 9E), and FRGPI was positively correlated with the TIDE score (Spearman: r = 0.300, *p* < 0.001; Figure 9F), indicating that patients with low FRGPI had better response and efficacy to ICI therapy. In addition, we verified the reliability of FRGPI in predicting the benefit of immunotherapy in patients with urothelial cancer who received anti-*PD-L1* immunotherapy in IMvigor210 cohorts. The low FRGPI group had a longer survival time after anti-*PD-L1* therapy than the high FRGPI group (Figure 9G), and the complete response (CR)/partial response (PR) group had lower FRGPI than the stable disease (SD)/progressive disease (PD) group (Figure 9H). The ROC curve suggested that FRGPI combined with neoantigen and TMB could predict the benefit of anti-*PD-L1* therapy more accurately (complex, AUC = 0.74; Figure 9I). The IC2+ level group, TC2+ level group, and inflamed phenotypes had the lowest FRGPI in terms of *PD-L1* expression level in immune cells (IC level), *PD-L1* expression level in tumor cells (TC level), and immune phenotype, respectively (Figures 9J–L). The above results indicate that patients with glioma and low FRGPI could be more sensitive to ICI therapy, especially anti-*PD-1* therapy.

### Potential Small Molecule Compounds Based on FRGPI

**FIGURE 10:**
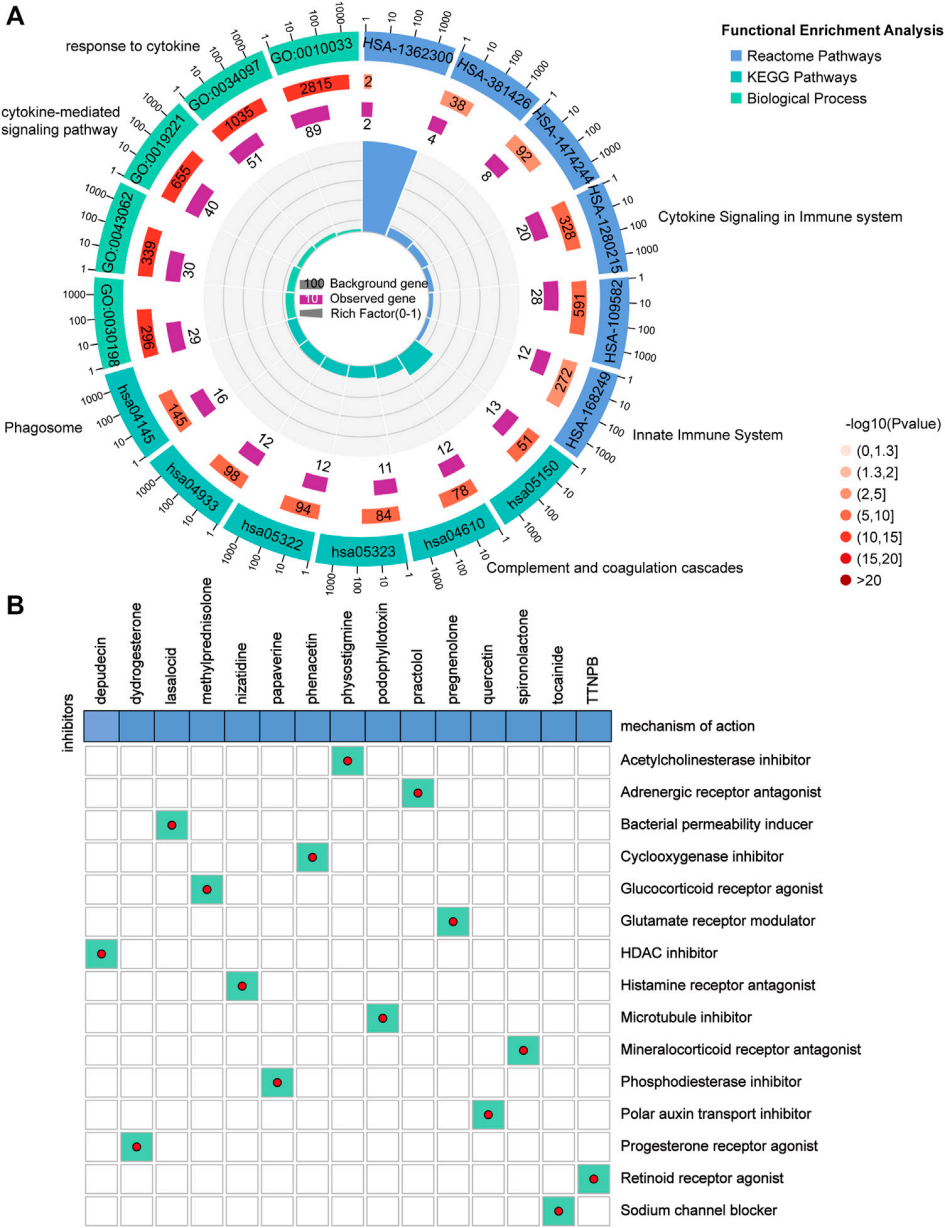
Potential small molecule compounds based on PRGPI. **(A)** Function enrichment analysis results of 263 DEGs between the high and low FRGPI groups. **(B)** Candidate small molecular drugs and mechanisms of action were discovered based on FRGPI. The abscissa represents the drugs, and the ordinate represents drug mechanisms of action.

The DEGs between the high and low FRGPI groups were further analyzed to identify potential small molecule compounds for glioma treatment. Consequently, 263 DEGs (Supplementary Table 9) were identified, including 184 upregulated and 79 downregulated genes (Supplementary Figure 3G). Functional enrichment analysis revealed that the DEGs were mainly enriched in response to cytokines, cytokine-mediated signaling pathways, cytokine signaling in the immune system, innate immune system, and phagosomes (Figure 10A; Supplementary Table 10). The DEGs were uploaded to the CMap database, and the underlying mechanisms of drug action were analyzed. As shown in Figure 10B, a total of 15 potential small molecule compounds (such as depactin, physostigmine, and phenacetin) and 15 drug action mechanisms (such as HDAC inhibitor, acetylcholinesterase inhibitor, cyclooxygenase inhibitor) were identified, which provide a reference to search for potential drugs for the treatment of glioma.

## Discussion

In this study, we successfully developed a ferroptosis-related gene prognostic index (FRGPI) to predict the sensitivity to temozolomide and the response to ICI therapy in patients with glioma. We comprehensively analyzed the role of FRGPI to identify different clinicopathological, molecular, and immune characteristics of patients with glioma that could improve treatment selection. From a new perspective of ferroptosis and glioma immune microenvironment regulation, our study can be used for more effective chemotherapy, immunotherapy, and targeted therapy plans for glioma.

Studies identified at least three major ferroptosis defense mechanisms have been identified in cells. Glutathione peroxidase 4 (GPX4) protects cells from ferroptosis by specifically catalyzing lipid peroxides in a glutathione-dependent manner (Seibt et al., 2019). Ferroptosis suppressor protein 1 (FSP1), locating on the cell membrane, prevents lipid peroxidation on the cell membrane and thus inhibiting ferroptosis by reducing ubiquinone (CoQ) to dihydroubiquinone (CoQH2) (Bersuker et al., 2019; Doll et al., 2019). Guanosine triphosphate cyclic hydrolase (GCH1), a rate-limiting enzyme for the synthesis of tetrahydrobiopterin (BH_4_), counteracts ferroptosis in a GPX4-independent manner (Kraft et al., 2020; Soula et al., 2020). Until Mao et al. discovered a new inhibitor of ferroptosis, dihydrowhey dehydrogenase (DHODH), which is independent of the classical GPX4 signaling pathway, and discovered the way of ferroptosis based on mitochondrial lipid peroxidation for the first time (Mao et al., 2021). Mao et al. proposed that there are at least three different subcellular localization of ferroptosis defense systems in cells: GPX4 in cytoplasm and mitochondria, FSP1 in plasma membrane, and DHODH in mitochondria, among which DHODH and GPX4 are the two main suppressors to defense against ferroptosis in mitochondrial (Mao et al., 2021). Tran et al. have demosrated GCH1 has an obvious ascending trend with grade increase, and plays a role in promoting progression in GBM (Tran et al., 2018), suggesting that GCH1 may play a more important role in high-grade glioma, while its role in low-grade glioma needs further discussion in the future. Our analysis showed that *GPX4*, *FSP1*, *GCH1*, and *DHODH* were more highly expressed in tumor tissue than in non-tumor brain tissue, especially in high-grade, IDH1 wild type, 1p19q non-codeletion gliomas, suggesting that glioma cells had a strong potential to resist ferroptosis. Identifying markers that could affect tumor immunity and ferroptosis status for the construction of FRGPI is pivotal. First, based on the expression profiles of FRGs, patients in TCGA cohort with glioma were divided into two ferroptosis-related subtypes (C1 and C2) with distinct differences in molecular and immune characteristics. *GPX4*, *FSP1*, *GCH1*, and *DHODH* were significantly upregulated in the patients of C1, implying that C1 represented a ferroptosis-suppressive status. Some genetic aberrations in glioma have been known for years, such as IDH1, TP53, MGMT, EGFR, ATRX, PTEN, AND CIC (Siegal, 2015; Weissmann et al., 2018; Ghosh et al., 2019). Our results showed that *IDH1*, *TP53*, *ATRX*, and *CIC* mutation frequencies in C2 were higher than in C1, but *PTEN* and *EGFR* in C1 were higher than in C2, suggesting that mutation frequencies of those genes may lead to different ferroptosis status. The patients in C1 had a worse overall prognosis than those in C2, indicating that identifying subtypes with different ferroptosis statuses had clinical prognostic implications. We then utilized WGCNA to identify candidate genes related to ferroptosis status and immunity. Afterward, we applied the “stepAIC” algorithm to construct the FRGPI based on four genes (*HMOX1*, *TFRC*, *JUN*, and *SOCS1*). The FRGPI proved to be an independent ferroptosis-related prognostic biomarker for glioma, with better survival in patients with FRGPI-low and worse survival in patients with FRGPI-high in both TCGA and CGGA cohorts.

FRGPI comprises four genes, *HMOX1*, *TFRC*, *JUN*, and *SOCS1*. Heme oxygenase 1 (*HMOX1*), a membrane-bound enzyme that cleaves the heme ring at the alpha-methene bridge to produce biliverdin, iron, and carbon monoxide, catalyzes the degradation of heme. Chang et al. discovered that *HMOX1* was a key mediator of BAY 11-7085 -induced ferroptosis that operated through cellular redox regulation and iron accumulation (Chang et al., 2018). Lu et al. (2019) discovered that the overexpression of HMOX1 enhanced both erastin- and RSL-3-triggered lipid reactive oxygen species to enhance ferroptosis. Arnold et al. (2014) observed that macrophages overexpressing *HMOX1* led to tumor immune suppression in pancreatic ductal adenocarcinoma. In addition, *HMOX1* was also observed to be related to immune suppression in neuroblastoma (Fest et al., 2016). The transferrin receptor (*TFRC*) encodes a cell surface receptor necessary for cellular iron uptake by receptor-mediated endocytosis. Wu et al. (2019) indicated that upregulation of *TFRC* promoted ferroptosis. Crepin et al. (2010) defined a novel class of fully human anti-*TFRC* antibodies suitable for immunotherapy against tumors whose proliferation depended on high levels of *TFRC* and iron uptake, such as myeloid leukemia and acute lymphoid. Jun proto-oncogene, AP-1 transcription factor subunit (*JUN*, also known as c-Jun) encodes a protein that is highly similar to the viral protein and interacts directly with specific target DNA sequences to regulate gene expression. A study observed that erastin could inhibit O-GlcNAcylation of *c-Jun* to suppress the malignant phenotypes of liver cancer cells (Chen et al., 2019). *C-Jun* has been shown to be related to T-cell proliferation and *PD-L1* expression (Jiang et al., 2013; Zdanov et al., 2016). The suppressor of cytokine signaling 1 (*SOCS1*) encodes a member of the *STAT*-induced *STAT* inhibitor, which functions downstream of cytokine receptors and takes part in a negative feedback loop to attenuate cytokine signaling. Saint-Germain et al. (2017) revealed that exogenous *SOCS1* was sufficient to regulate the sensitivity of cells to ferroptosis by reducing the expression of the cystine transporter *SLC7A11* and the level of glutathione. Saudemont et al. (2007) demonstrated that *SOCS1* expression restored via demethylation contributed to the resistance of tumor cells to CD8^+^ CTL-mediated killing. In the FRGPI calculation formula, the coefficients of *HMOX1*, *TFRC*, *JUN*, and *SOCS1* were positive. Therefore, there was a positive relationship between FRGPI, *HMOX1*, *TFRC*, *JUN*, and *SOCS1*, and they were promising therapeutic targets for glioma. Liu et al. (2020) established the ferroptosis potential index (FPI) based on 24 FRGs to explore the functional roles of ferroptosis and revealed that ferroptosis was associated with clinical features and the immune microenvironment in cancers. FRGPI was positively correlated with FPI, implying that FRGPI was a simpler and more convenient ferroptosis potential indicator in gliomas. In summary, FRGPI has been found to be a prognostic biomarker associated with cell sensitivity to ferroptosis and tumor immunity.

Screening patients with glioma who are good candidates for temozolomide chemotherapy and ICI therapy was a vital function of the FRGPI. Our data showed that patients with a high FRGPI were more sensitive to temozolomide treatment than those with a low FRGPI. Additionally, the low FRGPI group had a stronger response to ICI and better efficacy from ICI therapy than the group with a high FRGPI. Stemness, defined as the potential for self-renewal and differentiation from the cell of origin, is highly associated with tumor progression and chemoresistance (Shibue and Weinberg, 2017; Su et al., 2018; Cooper and Giancotti, 2019). Malta et al. (2018) developed stemness indexes (including mRNAsi and mDNAsi) to reveal intra-tumor molecular heterogeneity and indicated a strong relationship between immune microenvironment content and stemness. Our results showed that patients with a high FRGPI had lower mRNAsi but higher mDNAsi than those with a low FRGPI, indicating that FRGPI could affect chemoresistance and ICI response by altering tumor cell stemness. We also explored the correlation between the FRGPI and *PD-L1*, TMB, and MSI, which are well-known predictive biomarkers for immunotherapy. In general, *PD-L1*-positive cancers tended to respond to anti-*PD-1*/*PD-L1* therapies compared to *PD-L1*-negative cancers (Hansen and Siu, 2016). However, our results showed that the FRGPI had a negative ICI response but was positively related to the expression of *PD-L1*. *PD-L1* expression within the tumor microenvironment has predictive value for assessing response to anti-*PD-1*/*PD-L1* in many studies in melanoma (Weber et al., 2017), non-small-cell lung cancer (Fehrenbacher et al., 2016), and bladder cancer (Rosenberg et al., 2016) but this was not a consistent finding (Rosenberg et al., 2016; Sharma et al., 2017). Noticeably, tumors with negative *PD-L1* that escaped immune elimination were still sensitive to antibody-mediated *PD-L1* inhibitors (Noguchi et al., 2017). We hypothesized that the subcellular distribution of *PD-L1* in glioma tissues was more valuable than the *PD-L1* expression level measured by transcriptome data. Therefore, further studies are necessary to clarify the relationship between the subcellular distribution of *PD-L1* and the FRGPI. TMB and MSI have been recently evaluated as potential biomarkers for predicting ICI response in many cancer types (Bonneville et al., 2017; Yarchoan et al., 2017). Here, we found that the FRGPI had a significant correlation with TMB and MSI scores, which implied that TMB and MSI could explain why the FRGPI affected the response of patients with glioma to ICI, although there are other possible mechanisms involved in it.

The tumor immune microenvironment (TIME) is closely related to the progression, chemoresistance, and immunotherapy response (Cao and Yan, 2020; Hegde and Chen, 2020). Understanding the landscape of the TIME could help find new treatments for glioma or alter the TIME to improve the efficacy of immunotherapy. The infiltration of immune cells was distinct between the two subgroups. CD8^+^ T cells, resting and activated memory CD4^+^ T cells, regulatory T cells, resting NK cells, M0, M1, and M2 macrophages, activated myeloid dendritic cells, activated mast cells, memory B cells, and neutrophils were mainly enriched in the FRGPI-high subgroup with a worse prognosis, while naive CD4^+^ T cells, follicular helper T cells, activated NK cells, plasma B cells, naïve B cells, and monocytes were more common in the FRGPI-low subgroup with a better prognosis. Numerous studies have shown that dense infiltration of cytotoxic CD8^+^ T cells indicates a favorable prognosis (Bindea et al., 2013; Gentles et al., 2015; Fridman et al., 2017). M2 macrophages have been proven to be related to tumor growth, the development of an invasive phenotype, and are associated with a poor prognosis in breast, gastric, bladder, ovarian, and prostate cancers (Ruffell and Coussens, 2015). Conversely, M1 macrophages could signal a favorable prognosis in non-small-cell lung cancer, hepatocellular carcinoma, and ovarian and gastric cancers (Ruffell and Coussens, 2015). Our research results do not fully support these conclusions. Yin et al. found that high infiltrating levels of CD4^+^ T cells, B cells, CD8^+^ T cells, neutrophils, macrophages, and dendritic cells were all negatively correlated with the OS of lower-grade gliomas (Yin et al., 2020), which supports our study results. Considering that we did not use multiple algorithms to compare the immune cell infiltration landscape of glioma, we will further explore the relationship between the FRGPI and immune infiltration in future studies. Most importantly, the FRGPI could be of great significance for future researchers to develop an algorithm that specifically predicts the immune infiltration of glioma cells.

In addition, 184 upregulated and 79 downregulated genes were identified between the high and low FRGPI groups to explore the mechanism of action and potential small molecule compounds related to FRGPI. Functional enrichment analysis revealed that the FRGPI was significantly associated with immune-related pathways, including responses to cytokines, cytokine-mediated signaling pathways, cytokine signaling in the immune system, innate immune system, and phagosomes, which further indicated that tumor cell ferroptosis had a potential regulatory effect on tumor immunity. Finally, 15 potential small molecule compounds, such as depactin, physostigmine, and phenacetin, were predicted based on the FRGPI, which provided a reference for us to search for effective drugs to treat glioma.

Potential limitations of the present study are as follows. Firstly, a major limitation is that we use a public database rather than our own samples, so we will focus on collecting our own glioma samples to further verify the reliability of the research results. Secondly, the number of immunotherapy cohorts is limited, and it is extremely important to collect more ICI cohorts, especially for glioma, in the future. Finally, we did not explore the specific action mechanism of the FRGPI members, which will be an important direction of our future research.

## Conclusion

In conclusion, the FRGPI is a promising ferroptosis-related prognostic biomarker. It may help in distinguishing immune and molecular characteristics and accurately predicting the clinical outcome, possible temozolomide resistance, and ICI response in glioma. Hence, it is essential to systematically evaluate the FRGPI for each patient with glioma, which might assist oncologists to make decisions to administer ferroptosis-based anticancer therapy.

## Data Availability

The datasets presented in this study can be found in online repositories. The names of the repository/repositories and accession number(s) can be found in the article/Supplementary Material.

## Supplementary Material

Supplementary Figure 1 Aberrant expression of GPX4, GCH1, FSP1 and DHODH in glioma. **(A,B)** Boxplots of GPX4, FSP1, GCH1 and DHODH expression in different grade glioma samples in CGGA cohort. ns, no significance; **p* < 0.05; ***p* < 0.01; ****p* < 0.001. **(C–F)** Immunohistochemistry (IHC) staining for GPX4, FSP1, GCH1 and DHODH, respectively, in glioma and non-tumor brain tissue.

Supplementary Figure 2 Consensus clustering based on based on the expression of 94 ferroptosis suppressors. **(A)** CGGA validation set were divided into two different ferroptosis-related subgroups (G1 and G2) by consensus clustering based on based on 94 ferroptosis suppressors. **(B)** Kaplan–Meier analysis of patients in the two different ferroptosis-related subgroups. **(C)** Boxplots of the immuneScore and stromalScore between the two ferroptosis-related subtypes. **p* <  0.05, ***p* < 0.01, ****p* < 0.001. **(D)** Immune cell score heat map, where different colors represent the expression trend in each sample between two ferroptosis-related subgroups. **p* < 0.05, ***p* < 0.01, ****p*  < 0.001. **(E)** The percentage abundance of tumor infiltrating immune cells in each sample, with different colors and different types of immune cells. The abscissa represents the sample, and the ordinate represents the percentage of immune cell content in a single sample.

Supplementary Figure 3 Screening and verification of the hub gene used to construct FRGPI. **(A)** Differential gene expression heatmap, where different colors represent expression trends in different tissues. Due to the large number of differential genes, the 50 up-regulated genes and 50 down-regulated genes with the largest differential changes are shown here. Red, up-regulated; blue, down-regulated. **(B)** Correlation of weighted gene correlation network analysis (WGCNA) divided all protein-coding genes into 10 modules. **(C)** The protein-protein interaction network of the 7 hub genes related both ferroptosis and tumor immunity. **(D)** Principal component analysis (PCA) plot of glioma samples based on HMOX1, TFRC, JUN and SOCS1 expression profiles in CGGA validation set. **(E,F)** GSVA analysis results of the relationship between FRGPI and PI3K_AKT_MTOR_SIGNALING (Spearman r = 0.660, *p* < 0.001) and MTORC1_SIGNALING (Spearman r = 0.810, *p* < 0.001).

Supplementary Figure 4 The validation of the prognosis predicting value of FRGPI in CGGA validation set. **(A)** FRGPI, survival status and HMOX1, TFRC, JUN and SOCS1 expression profiles of each sample are shown. **(B)** Kaplan–Meier analysis of glioma patients with low or high FRGPI. **(C)** ROC curves validate the prognostic value of FRGPI in glioma patients (1 year AUC: 0.86, 3 years AUC: 0.88, 5 years AUC: 0.84).

Supplementary Figure 5 The prediction accuracy of FRGPI in different clinical character groups. (**A–H)** The prediction accuracy of FRGPI in <45- and >45- year old group, LGG and GBM group, IDH1-WT and IDH1-Mut group, 1p19q codel and 1p19q non-codel group in TCGA training set. (**I–P)** The prediction accuracy of FRGPI in <45- and >45- years group, LGG and GBM group, IDH1-WT and IDH1-Mut group, 1p19q codel and 1p19q non-codel group in in CGGA validation set.

Supplementary Table 1 The baseline clinical characteristics of glioma patients.

Supplementary Table 2 24 ferroptosis-related genes for consensus clustering.

Supplementary Table 3 173 ferroptosis-related genes list.

Supplementary Table 4 1793 immune-related genes list.

Supplementary Table 5 94 ferroptosis suppressors list.

Supplementary Table 6 2183 DEGs list between C1 and C2.

Supplementary Table 7 2189 DEGs list between G1 and G2.

Supplementary Table 8 2613 genes list in blue module.

Supplementary Table 9 263 DEGs between the high and low FRGPI groups.

Supplementary Table 10 Function enrichment analysis results for 263 DEGs.
