# Supplementary figures and images for: A Ferroptosis-Related Gene Prognostic Index to Predict Temozolomide Sensitivity and Immune Checkpoint Inhibitor Response for Glioma
Source: Front Cell Dev Biol. 2022 Jan 31;9:812422. doi: 10.3389/fcell.2021.812422 (PMC8842730; doi:10.3389/fcell.2021.812422)

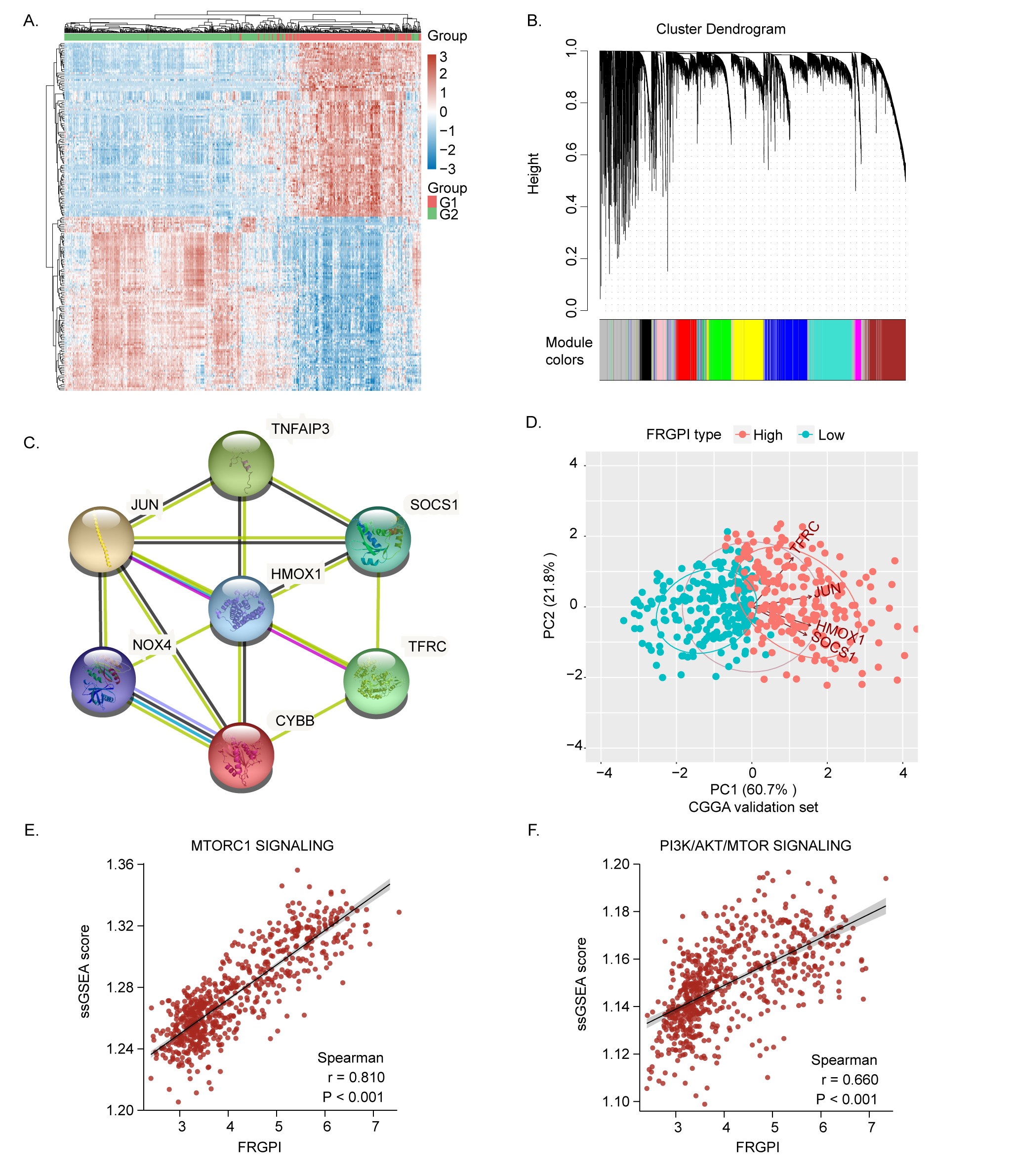

Supplement: Supplementary file 1 [file Image3.TIF]

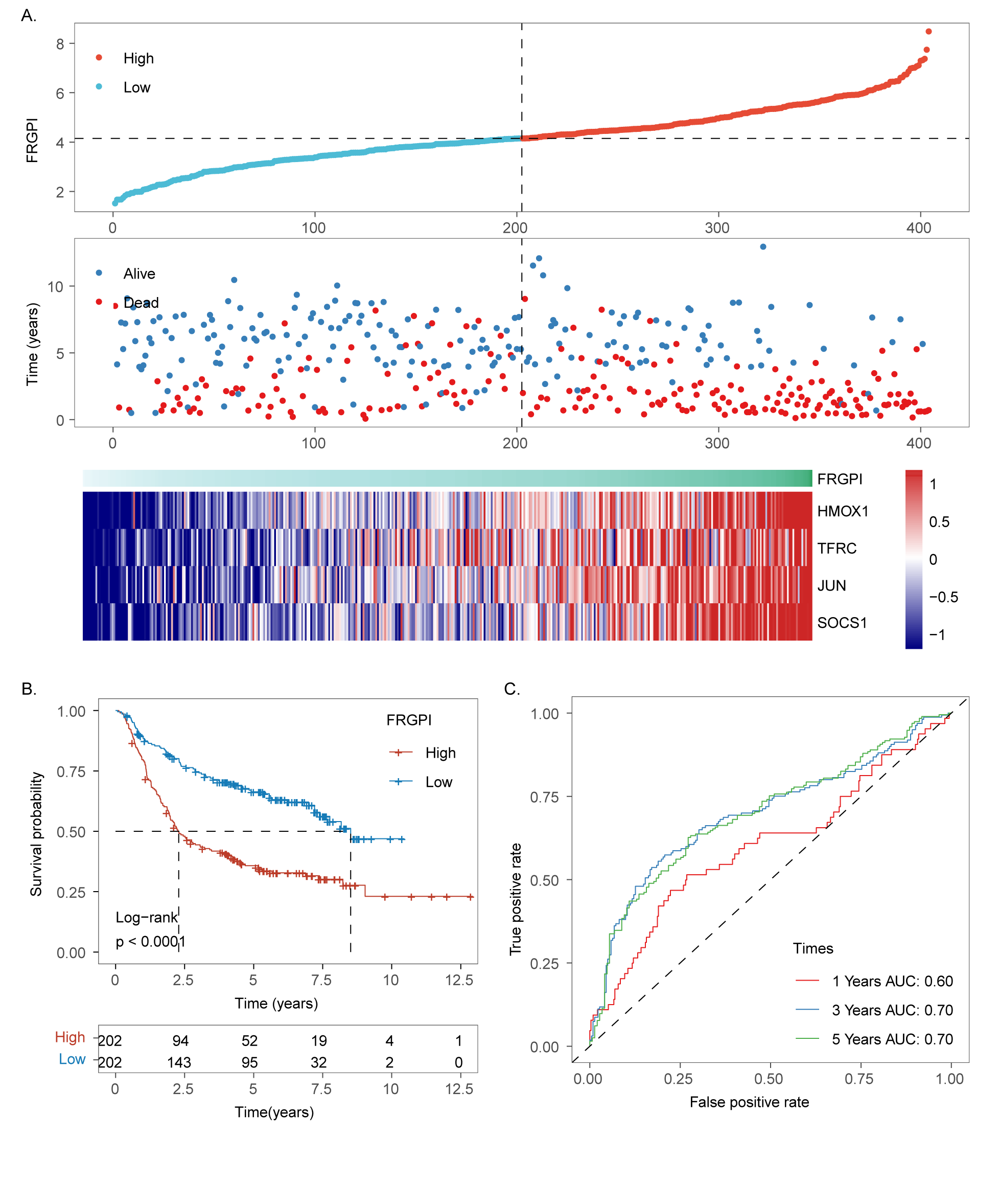

Supplement: Supplementary file 2 [file Image4.TIF]

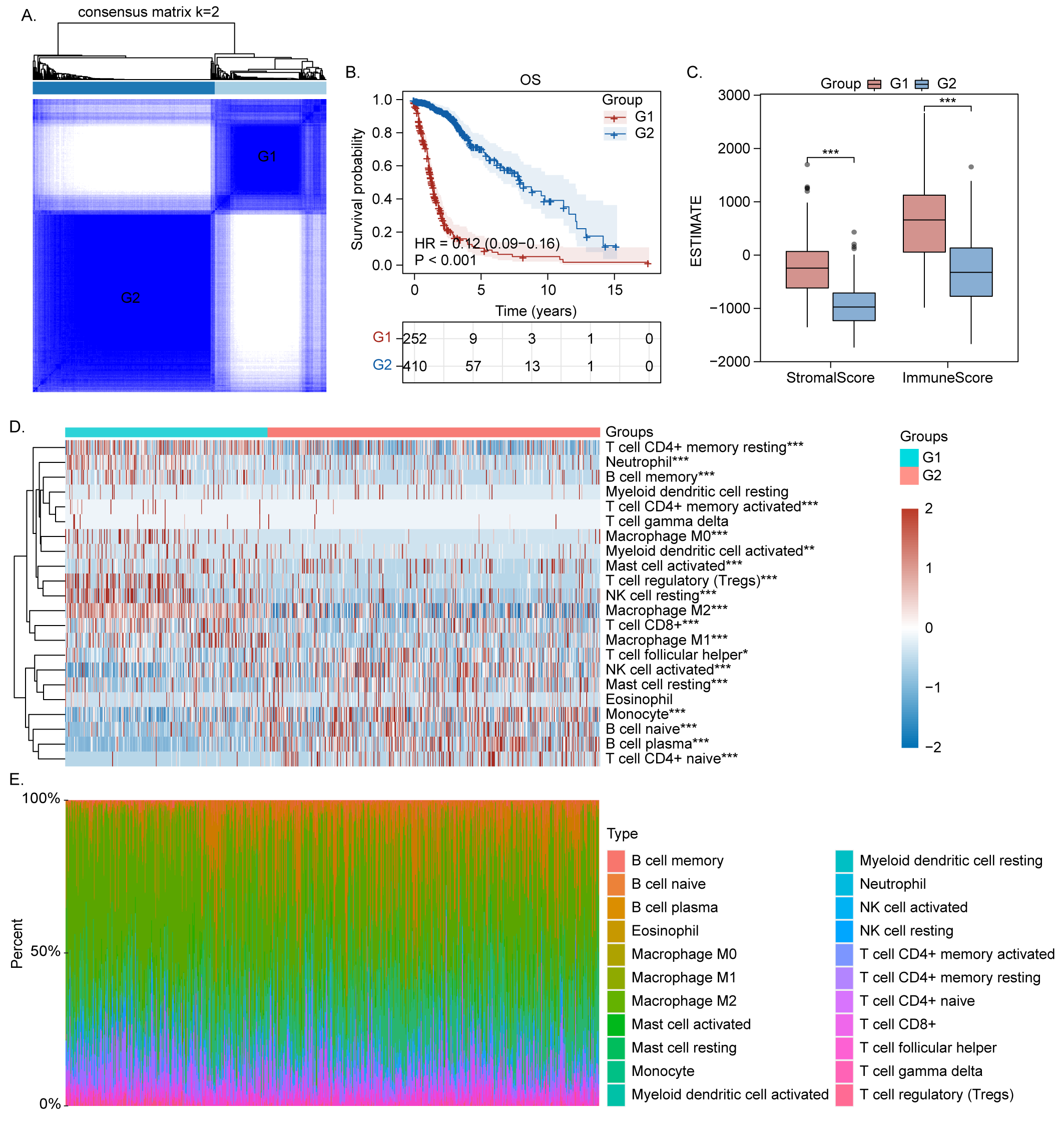

Supplement: Supplementary file 3 [file Image2.TIF]

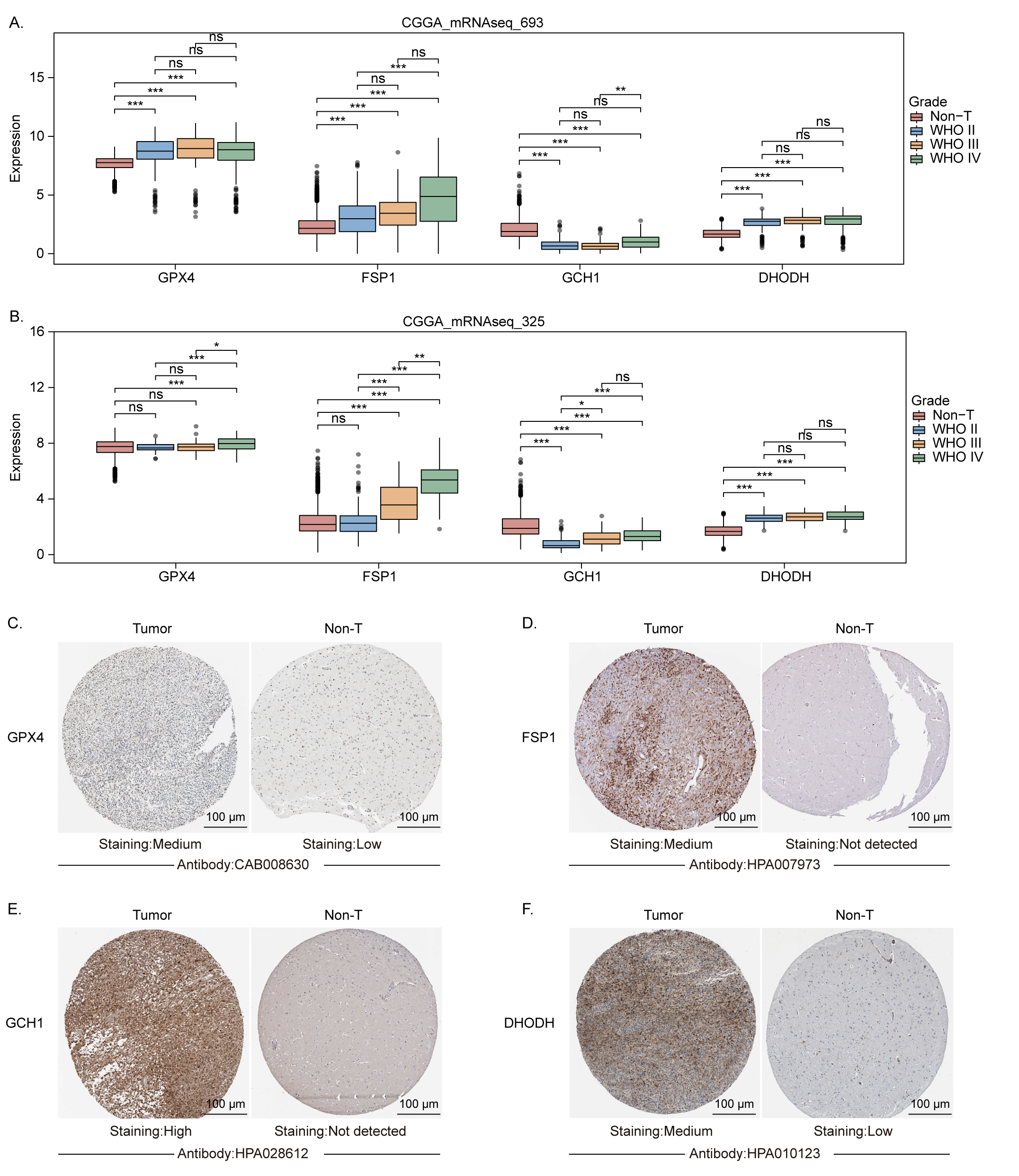

Supplement: Supplementary file 4 [file Image1.TIF]

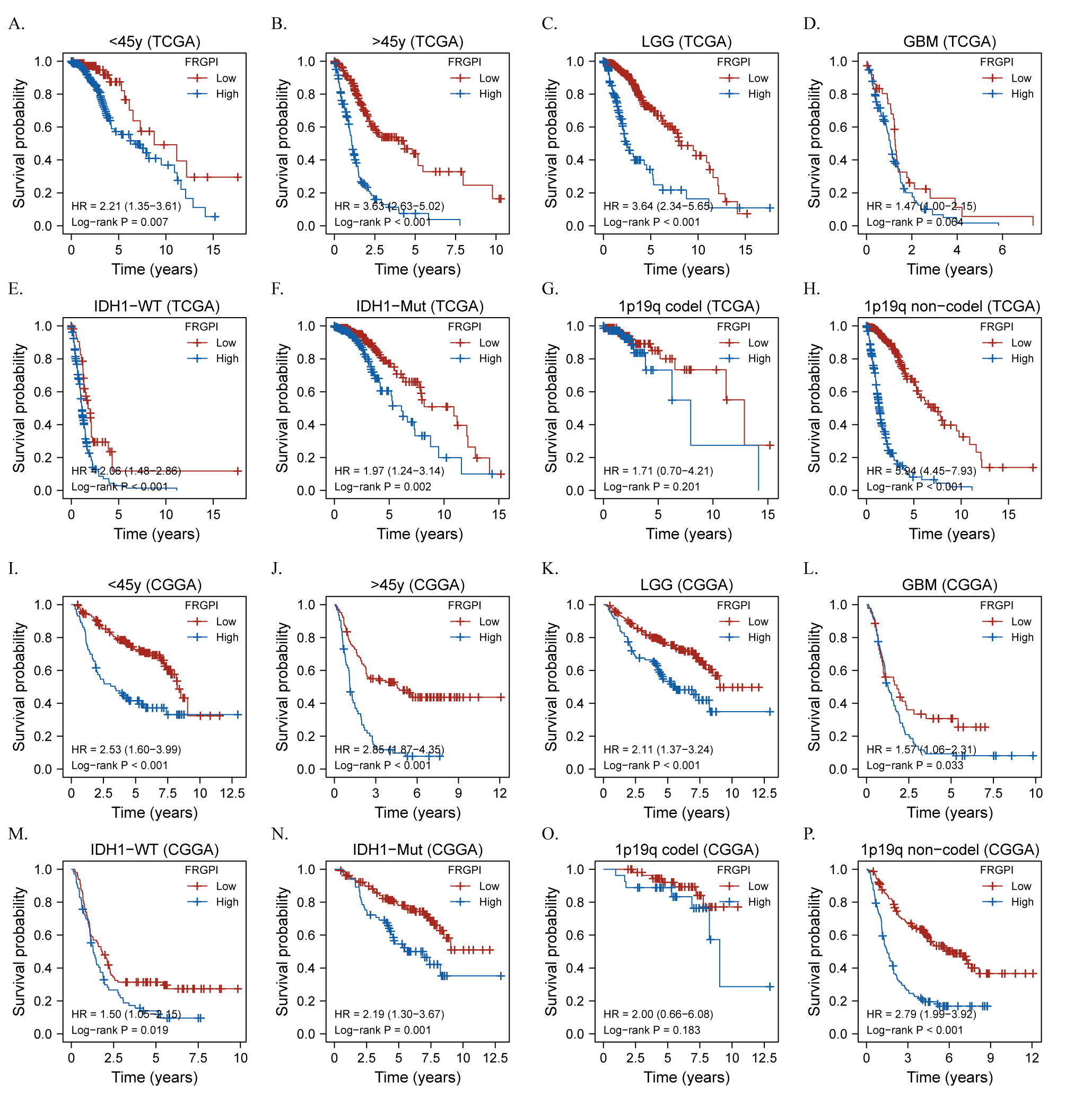

Supplement: Supplementary file 5 [file Image5.TIF]
